# *Physcomitrella patens* Has *Kinase-LRR R* Gene Homologs and Interacting Proteins

**DOI:** 10.1371/journal.pone.0095118

**Published:** 2014-04-18

**Authors:** Yusuke Tanigaki, Kenji Ito, Yoshiyuki Obuchi, Akiko Kosaka, Katsuyuki T. Yamato, Masahiro Okanami, Mikko T. Lehtonen, Jari P. T. Valkonen, Motomu Akita

**Affiliations:** 1 Department of Biotechnological Science, Kinki University, Wakayama, Japan; 2 Department of Agricultural Sciences, University of Helsinki, Helsinki, Finland; University of California – Davis, United States of America

## Abstract

Plant disease resistance gene (*R* gene)-like sequences were screened from the *Physcomitrella patens* genome. We found 603 kinase-like, 475 Nucleotide Binding Site (NBS)-like and 8594 Leucine Rich Repeat (LRR)-like sequences by homology searching using the respective domains of PpC24 (Accession No. BAD38895), which is a candidate *kinase-NBS-LRR* (*kinase-NL*) type *R*-like gene, as a reference. The positions of these domains in the genome were compared and 17 kinase-NLs were predicted. We also found four TIR-NBS-LRR (TIR-NL) sequences with homology to *Arabidopsis* TIR-NL (NM_001125847), but three out of the four TIR-NLs had tetratricopeptide repeats or a zinc finger domain in their predicted C-terminus. We also searched for kinase-LRR (KLR) type sequences by homology with rice OsXa21 and *Arabidopsis thaliana* FLS2. As a result, 16 KLRs with similarity to OsXa21 were found. In phylogenetic analysis of these 16 KLRs, PpKLR36, PpKLR39, PpKLR40, and PpKLR43 formed a cluster with OsXa21. These four PpKLRs had deduced transmembrane domain sequences and expression of all four was confirmed. We also found 14 homologs of rice OsXB3, which is known to interact with OsXa21 and is involved in signal transduction. Protein–protein interaction was observed between the four PpKLRs and at least two of the XB3 homologs in Y2H analysis.

## Introduction

Plants are frequently exposed to pathogens, against which they can protect themselves in several ways, e.g., specific recognition and subsequent expression of disease response genes, accumulation of antibiotics, and elimination of the pathogen and infected tissue by programmed cell death. These responses are triggered by so-called disease resistance genes (*R* gene products). The R proteins recognize virulence gene products of specific pathogens. *R* genes can be categorized into several groups [1], [2]. The most abundant group comprises the Nucleotide Binding Site (NBS) and Leucine Rich Repeat (LRR) (NL) types, which are divided into groups by their N-terminal domains: Toll/interleukin-1 like receptor (TIR) or coiled coil (CC). The second group is called the KLR type and has intracellular kinase, transmembrane (TM) and extracellular LRR domains. The third and fourth groups are composed only of intracellular kinases or membrane-anchored LRRs. *KLR* type *R* gene products are predicted to be receptors and the rice protein *Oryza sativa Xanthomonas* resistance 21 (OsXa21) is one of the best studied examples. OsXa21 was the first kinase-LRR (KLR) type *R* gene that was found in *Oryza sativa* [3] and recognizes OsAx21 (activator of OsXa21-mediated immunity) from *Xanthomonas oryzae* pv. *oryzae* [4]. It was reported that OsXa21 and OsAx21 are in the relation between so-called pattern recognition receptors (PRRs) and pathogen associated molecular patterns (PAMPs) [5]. KLR products are predicted to transduce signals through their Ser/Thr kinase activity [6]. Two types of kinase domains, non-RD (arginine aspartate) and RD kinases, are found in KLRs [7]. RD kinases, e.g., CERK1 [8] of the chitosan receptor and *Arabidopsis thaliana* BAK1 [9], have characteristic RD residues at the activation site (R). RD kinases are regulated by autophosphorylation [7]. Dardick *et al*. [10] showed that another group of KLRs have a non-RD motif, e.g., OsXa21 and *A. thaliana* flagellin sensitive 2 (FLS2) [11]. The kinase domains of OsXa21 and AtFLS2 have autophosphorylation function [12], [13]. In rice, Xa21 binding protein 3 (OsXB3), which is an E3 ubiquitin, is suggested to be involved in the OsXa21 signaling pathway. OsXB3 has a highly conserved ankyrin repeat domain and zinc finger domain. When OsXa21 recognizes pathogens, the kinase domain may interact with OsXB3 and induce signal transduction [14]. However, the functions of KLRs in disease resistance are largely unclear.

To understand the mechanisms of the plant disease response, we have used a moss (*Physcomitrella patens*) as a model plant. This moss has significant advantages for biological and evolutionary studies. Specifically, a high-quality whole genome sequence is available for this moss and information on the functions of its genes has been quickly accumulated [15]. Although the disease responses of mosses have begun to be studied only recently, saprobic and parasitic interactions have been reported between mosses and fungi, e.g., *Hylocomium splendeus* is infected by the saprobic and parasitic fungi *Coniochaeta velutina* [16]. Pathogenic fungi were isolated from *Racomitrium japonicum* and these fungi were also able to infect higher plants and *P. patens* [17]. *P. patens* is also infected by *Erwinia carotovora* ssp. *carotovora* and *Botrytis cinerea* [18], [19]. When *P. patens* is infected by *Pythium*, several disease response related genes are induced [19]. We have also reported that *P. patens* responds to chitosan elicitors and releases peroxidase [20], [21]. Regarding *R* genes, we reported that *P. patens* possibly had NL-type genes (*PpC24*, AB078154) [22]. PpC24 has the NBS domains of *TIR-*type *R* genes, but the N-terminus is a Ser/Thr kinase not a TIR. Wide sequence analysis indicated that *P. patens* also has *TIR-NL-* and *CC-NL-*type *R* gene homologs [23], [24]. However, information about *KLR-*type *R* gene homologs in mosses has to the best of our knowledge not been reported to date.

The aim of this study was to analyze *R* gene homologs in *P. patens*. We found many kinase-like, NBS-like, and LRR-like sequences in the *P. patens* genome. The relative positions of these domains in the genome were analyzed and we predicted the existence of kinase-LRRs in this moss. We also found OsXa21 homologs. By phylogenetic analysis, we detected four kinase-LRR sequences that showed high similarity to OsXa21. These four KLR homologs had TM domains like OsXa21. We also found that the moss had homologs of the OsXa21-interacting protein (OsXB3) and that these homologs could interact with the KLR homologs of *P. patens*.

## Materials and Methods

### Plant material

*P. patens* ecotype Gransden Wood was grown in Petri dishes (diameter 9 cm) on cellophane membranes (#300, RENGO, Osaka, Japan) placed on agar BCD medium [1 mM MgSO_4_, 1.85 mM KH_2_PO_4_ (pH 6.5, adjusted with KOH), 10 mM KNO_3_, 45 µM FeSO_4_, 0.22 µM CuSO_4_, 0.19 µM ZnSO_4_, 10 µM H_3_BO_4_, 0.10 µM Na_2_MoO_4_, 2 µM MnCl_2_, 0.23 µM CoCl_2_, 0.17 µM KI] [25] supplemented with 1 mM CaCl_2_, 45 µM ethylenediaminetetraacetic acid disodium salt (Na_2_-EDTA), and 5 mM ammonium tartrate, and solidified with 0.8% agar. The cultures were grown at 23°C with continuous light (ca.100 µmol m^−2^ s^−1^ (PFD)).

### Extraction of RNA, cDNA synthesis, and PCR

Total RNA was extracted from 200 mg (FW) *g*ametophore tissue using an Agilent Plant RNA Isolation Mini Kit (Agilent Technologies, Santa Clara, CA, USA) according to the manufacturer's instructions. The RNA was treated with recombinant DNase I (RNase-free; Takara, Otsu, Japan). After phenol extraction and ethanol precipitation, cDNA was synthesized from 1 µg total RNA using ReverTra Ace (Toyobo, Osaka, Japan). PCR analysis of the cDNA was performed using Herculase II Fusion DNA Polymerase (Agilent Technologies) in a total final reaction volume of 15 µL. GoTaq Green Master Mix (Promega, Fitchburg, WI, USA) was used for insert checks of transgenic *Escherichia coli*. Reaction mixtures (8 µL) contained 4 µL GoTaq DNA Polymerase, 0.5 µL each primer (5 µM), and 3 µL of cell suspension as the template. Primers for each PCR were designed using Primer-BLAST and primer3 plus (http://www.bioinformatics.nl/cgi-bin/primer3plus/primer3plus.cgi) (Table S1).

### Search for homologous genes

Genomic sequence of *P. patens* was obtained from the Phytozome database (http://www.phytozome.net/). Homologous gene searches of the *P. patens* genome were performed using the BLAST system. Kinase and LRR domains were searched using BLASTP with kinase, NBS and LRR sequences of PpC24 (BAD38895). Sequences in which the distance between each domain was 2 kbp or less were selected and recorded as PpKNLs (kinase-NBS-LRR domains) or PpKLRs (kinase-LRR domains). In addition, kinase-LRR sequences were predicted by the homolog search program of Phytozome against OsXa21 (U37133) and AtFLS2 (NP_199445).

### Sequence analysis

DNA sequences were determined using a CEQ2000XL (Beckman Coulter, Brea, CA, USA) following the company's instructions. Some DNA sequences were entrusted to Operon Biotechnologies Inc. (Ota, Tokyo, Japan). The data were analyzed using the Genetyx version 10 software (Genetyx, Tokyo, Japan). Sequence alignment and phylogenetic analysis were carried out using the ClustalW program in Genetyx. Phylogenetic analysis of amino acid sequences was also carried out using the ClustalW program available at DDBJ (http://www.ddbj.nig.ac.jp/). A phylogenetic tree was built using Dendroscope. TM domains of the PpKLRs were predicted using the SOSUI program (http://bp.nuap.nagoya-u.ac.jp/sosui/).

### Yeast two hybrid assay

Y190 strain yeast was grown on YPD medium [10 g/L yeast extract (Nakalai tesque, Kyoto, Japan), 20 g/L peptone (Nakalai tesque), and 20 g/L d-glucose (Nakalai tesque)] [26]. Co-transformants were grown on SD-W, L medium (6.7 g/L Difco™ Nitrogen Base w/o Amino Acids (Becton, Dickinson and Company, Franklin Lakes, NJ, USA) 2 g/L d-Glucose, 0.002% adenine, 0.002% uracil, 0.03% Lys, 0.01% His) and selected on SD-W, L, H medium (0.01% His in SD-W, L) containing 40 mM 3-Amino-1,2,4-triazole (3-AT).

Predicted kinase domain sequences of *PpKLR36*, *PpKLR39*, *PpKLR40*, and *PpKLR*43 were amplified by PCR from cDNA and these PCR products were cloned into the HincII site of the pBlueScript SK plasmid. Primers were designed according to sequence data from the Phytozome database (Table S1). The plasmids were digested with PspXI/EcoRV and the fragments were cloned into the pGBT9 vector (Clontech) at the PstI site. RT-PCR products of *PpXB3s* were cloned into the pGAD10 vector (Clontech) at the EcoRI site. The vectors were transformed into yeast strain Y190 and the yeasts were incubated on SD-W, L plates at 27°C. The transformed yeasts were incubated overnight at 27°C in YPD liquid medium. After centrifugation at 1000×g, the pellets were washed twice with SD-W, L, H liquid medium. After suspension in SD-W, L, H liquid medium, the turbidity was adjusted to 0.2 at OD_600_. These suspensions were dropped on SD-W, L, H plates and incubated at 27°C.

## Results

### *P. patens* has NL, KLR-type genes

*PpC24* was predicted to be a *kinase*-*NBS* (Pp1s4_271V6.1) gene but a *LRR* sequence (Pp1s4_269V6.1) was found at the C-terminus adjacent region in the genome sequence. We detected that long transcripts were transcribed through PpC24 (AB078154) including several splicing variants, but all of them had a stop codon at the C-terminal end of the NBS.

BLAST analysis using these putative *kinase*, *NBS* and *LRR* sequences revealed 603 *kinase*, 475 *NBS* and 8594 *LRR*-like sequences in the moss genome. The distances between each domain are commonly less than 500 bp in *R* genes and their homologs; e.g. *PpC24*+*LRR* (described above), *Nicotiana tabacum N* (BAD12594) and *TIR*-*NL* of *A. thaliana* (At5G44510 and NM_001125847). Since the possibility of splicing had not been eliminated, we compared the positions of the predicted coding regions and collected sets of tentatively predicted *kinase*-*NBS*-*LRR* gene sequences in which the distance between each domain was less than 2 kbp. As a result, we found 17 *kinase*-*NLs* (Table 1). The distances between each domain were less than 780 bp in the genome. The kinase and NBS domains of these 17 kinase-NLs had conserved Ser/Thr kinase and TIR-type NBS sequences. *Kinase*-*NL*-type genes such as PpC24 [22] have been reported in moss [23] and also as an *R* gene of wheat [27], although the function of the wheat *R* gene is not clear. We also found four *TIR*-*NL* sequences (Table 2) by BLAST analysis using *A. thaliana* TIR-NL (NM_001125847) as the query. The TIR-NL sequences had tetratricopeptide repeats (TNL1 and 2) or a zinc finger domain (TNL3) in their C-terminus. These structures could be unique because no *R* genes having these domains in the C-terminus have been reported. However, TNL4 had a premature stop codon at the 3′-end of the NBS, indicating that a TIR-NBS transcript could be produced.

**Table 1:** Locations of predicted *Kinase-NL* genes in the *P. patens* genome.

					Gene ID		
	Scaffold	Location			Kinase	NBS	LRR
*PpKNL1*	4	1738811	-	1743286	Pp1s4_271V6.1 2)		Pp1s4_269V6.1
*PpKNL2*	107	727824	-	731384	Pp1s107_101V6.1	Pp1s107_101V6.2	Pp1s107_101V6.3
*PpKNL3*	113	993176	-	997774	Pp1s113_185V6.1 1)		
*PpKNL4*	158	673509	-	677386	Pp1s158_132V6.1 2)		Pp1s158_133V6.1
*PpKNL5*	158	699611	-	703907	Pp1s158_140V6.1 2)		Pp1s158_139V6.1
*PpKNL6*	17	1526214	-	1532049	Pp1s17_234V6.1 1)		
*PpKNL7*	17	1840397	-	1844127	Pp1s17_276V6.1	Pp1s17_276V6.2	Pp1s17_277V6.1
*PpKNL8*	180	434595	-	437932	Pp1s180_59V6.1 2)		Pp1s180_60V6
*PpKNL9*	180	685108	-	691200	Pp1s180_101V6.1 1)		
*PpKNL10*	2	1242958	-	1246400	Pp1s2_244V6.1	Pp1s2_245V6.1	Pp1s2_246V6.1
*PpKNL11*	223	477323	-	482156	Pp1s223_84V6.1	Pp1s223_83V6.1	Pp1s223_81V6.1
*PpKNL12*	37	146900	-	150243	Pp1s37_18V6.1 2)		Pp1s37_19V6.1
*PpKNL13*	37	325178	-	328605	Pp1s37_60V6.1 2)		Pp1s37_59V6.2
*PpKNL15*	396	165625	-	170945	Pp1s396_25V6.1 2)		Pp1s396_28V6.1
*PpKNL16*	40	1047192	-	1051275	Pp1s40_165V6.1 2)		Pp1s40_166V6.1
*PpKNL17*	508	639	-	6531	Pp1s508_5V6.1 2)		Pp1s508_2V6.1
*PpKNL18*	68	286024	-	289607	Pp1s68_70V6.1 2)		Pp1s68_69V6.1

The numbers indicate the start of putative kinase domain regions and the end of LRR regions in the *P. patens* genome. Scaffold numbers and locations were obtained from the Phytozome database (Ver 9.1). *PpKNL1* includes the *PpC24* sequence (AB078154, [22]).

1) Annotated as a single sequence (kinase-NBS-LRR).

2) Annotated as a kinase-NBS sequence.

**Table 2:** Locations and characteristics of *TIR-NL* genes in the *P. patens* genome.

						Gene ID	
	Scaffold	Location			C-terminal domain (Length in aa.)	TIR-NBS	LRR
TNL1	162	975509	-	981603	Tetratricopeptide repeat (213)	Pp1s162_170V6.11)	
TNL2	231	538859	-	543296	Tetratricopeptide repeat (68)	Pp1s231_60V6.11)	
TNL3	145	564173	-	569187	Zinc finger (44)	Pp1s145_93V6.11)	
TNL4	32	2201702	-	2207173		Pp1s32_318V6.1	Pp1s32_318V6.2

The numbers indicate the putative TIR-NL regions in the *P. patens* genome. Scaffold numbers and locations were obtained from the Phytozome database (Ver 9.1). C-terminal domains were predicted by Pfam.

1) Annotated as a single sequence (TIR-NBS-LRR).

**Figure 1:**
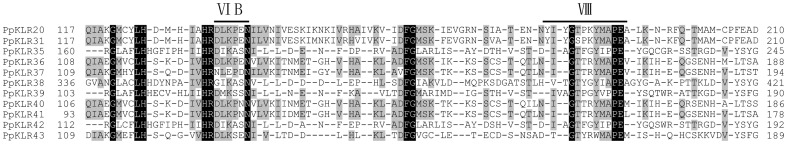
Alignment of conserved Ser/Thr kinase regions of the PpKLRs. The amino acid sequences were predicted from the Phytozome database (See Table 2). Bars indicate conserved Ser/Thr kinase regions: sub-domain VIB (DLKXXN) and sub-domain VIII (G(T/S)XX(Y/F)XAPE) [32]. The numbers indicate the putative kinase domain positions in the aa sequence. Shaded residues indicate similar amino acids.

**Figure 2:**
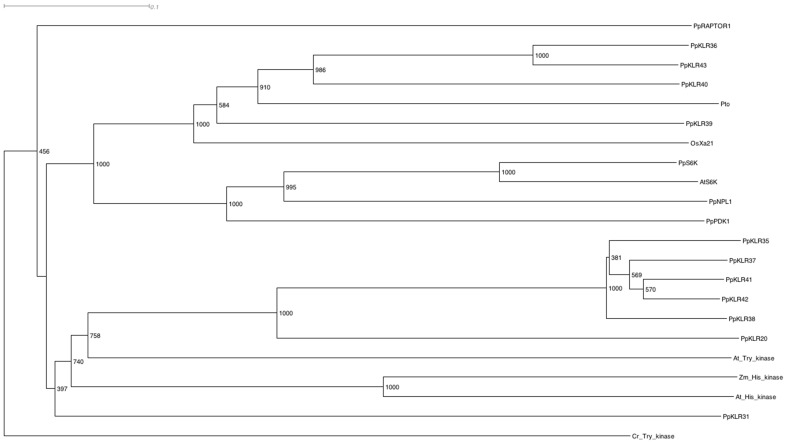
Phylogenetic analysis of PpKLR kinase regions and other plant kinases. The kinase domain sequences of PpKLRs that were suggested to be CDSs in Phytozome (Ver 9.1) were compared with known plant kinases (Table S3) including Ser/Thr, Tyr, and His kinases. A *Chlamydomonas* kinase (XP_001697921) was used as an outgroup. Phylogenetic trees were constructed by the neighbor-joining method. Numbers at the nodes indicate bootstrap values from 1,000 replicates.

**Figure 3:**
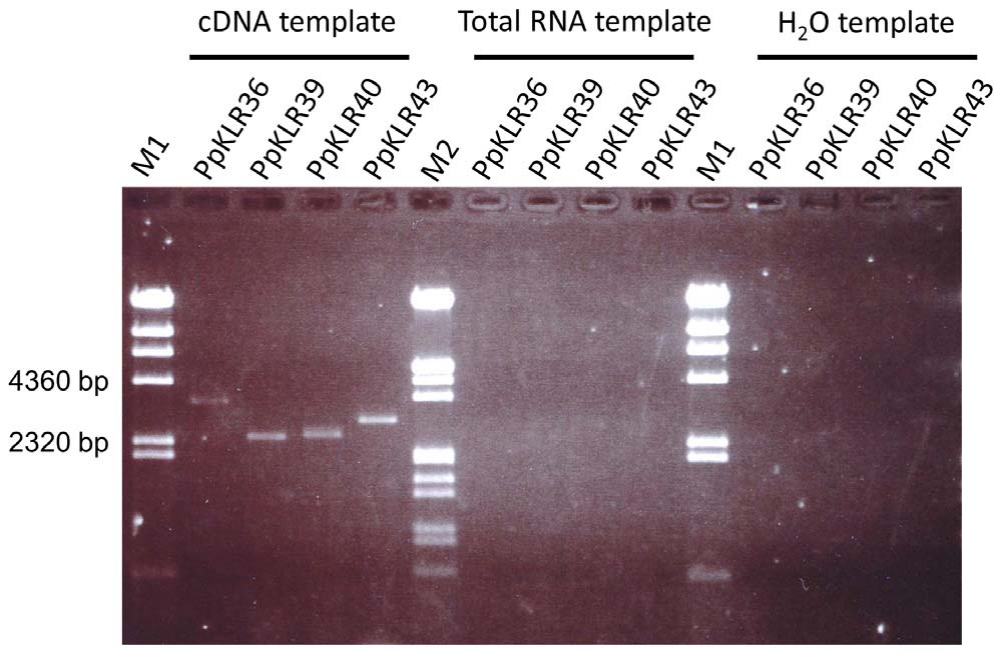
Expression of the four PpKLRs. Total RNA was extracted from gametophyte tissues and treated with DNase before cDNA was prepared. NC: negative control (H_2_O). Primers were designed according to sequence data from the Phytozome database (see Table S1).

Analysis of the 603 putative kinases indicated that 29 of them had conserved Ser/Thr kinase sequences as PpC24 (Table S2). We then searched for sequences in which the kinase domain was adjacent to a putative LRR domain by applying a similar strategy used to search for KNLs. We checked the orientation of each domain and/or the existence of stop codons and finally predicted no kinase-LRR sequences by this strategy. We also searched for KLRs based on similarity with OsXa21and AtFLS2. Whereas no sequences similar to the AtFLS2 kinase were found, the BLAST results indicated that there were 45 KLR homologs of OsXa21 (PpKLR1-PpKLR45). However, 34 OsXa21 homologs showed low similarity in each domain and/or had not been annotated in the Phytozome or NCBI databases as kinase-LRRs. We then checked whether these unannotated OsXa21 homologs were expressed using RT-PCR. We found that the PpKLR25 (AB872938) and PpKLR45 (AB872937) sequences were transcribed, but two or more stop codons were predicted in all frames. Therefore, these PpKLRs were excluded from further study. Finally, we analyzed 11 OsXa21 homologs from the *P. patens* genome (Table 3). Note that the length of the LRR domain in these 11 sequences varied significantly (see below). Figure 1 shows the conserved regions predicted to be kinase domains in the PpKLRs. We also tried to search for RLK5-type LRR-RLK candidates of counterpart peptides that had a characteristic structure in which the length was shorter than the LRR [6], but we found no RLK5-type genes. The relationship between the putative amino acid sequences of the kinases in the PpKLRs and other plants kinases was phylogenetically analyzed. An *A. thaliana* kinase (XP_002983016) and His and Tyr kinases of *Zea mays*, (NP_56527) and (NP_566335), respectively, formed different clusters from the PpKLRs. PpKLR31 formed a single root. Other PpKLRs, except for PpKLR36 (Pp1s172_87V6.1), PpKLR39 (Pp1s247_8V6.1), PpKLR40 (Pp1s27_27V6.1) and PpKLR43 (Pp1s48_171V6.1), formed clusters with other plant genes (Figure 2). PpKLR36, PpKLR39, PpKLR40 and PpKLR43 branched earlier than the other PpKLRs and plant genes and formed a cluster with OsXa21 and P. s. tomato (Pto) [28]. Since this result indicated that the relationships of PpKLR36, PpKLR39, PpKLR40, and PpKLR43 to OsXa21 were stronger than those of the other PpKLRs, we focused on these four PpKLRs. Their expression was confirmed by RT-PCR (Figure 3). Since the target sequences of the RT-PCR were designed to span the two domains, PpKLR36, PpKLR39, PpKLR40 and PpKLR43 could all be expressed as a single sequence, as predicted in the Phytozome database.

**Table 3:** Location of predicted kinase and LRR domains of PpKLRs in the *P. patens* genome.

		Location						Gene ID	
	Scaffold	Kinase			LRR			Kinase	LRR
*PpKLR20*	345	337817	-	336981	335332	-	332582	Pp1s345_31V6.1	Pp1s345_29V6.1
*PpKLR31*	68	198249	-	199056	195237	-	197808	Pp1s68_46V6.1 1)	
*PpKLR35*	169	489497	-	487952	487661	-	489131	Pp1s169_92V6.1 1)	
*PpKLR36*	172	834678	-	835482	835629	-	838500	Pp1s172_87V6.1 1)	
*PpKLR37*	176	665895	-	666693	665664	-	662502	Pp1s176_100V6.1 1)	
*PpKLR38*	202	591336	-	590535	594675	-	591,561	Phpat.017G029400.1 1)	
*PpKLR39*	247	110240	-	112019	110642	-	112064	Pp1s247_8V6.1 1)	
*PpKLR40*	27	152621	-	153422	150224	-	151370	Pp1s27_27V6.1 1)	
*PpKLR41*	374	35274	-	35973	36177	-	38088	Pp1s374_11V6.1 1)	
*PpKLR42*	385	215904	-	215100	214803	-	213585	Pp1s385_25V6.1 1)	
*PpKLR43*	48	1252036	-	1252843	1248898	-	1251535	Pp1s48_171V6.1 1)	

Numbers indicate the putative start and end of each domain in the *P. patens* genome. Scaffold numbers and locations were obtained from the Phytozome database (Ver 9.1).

1) Annotated as a kinase-LRR single sequence.

### Structure of PpKLRs

**Figure 4:**
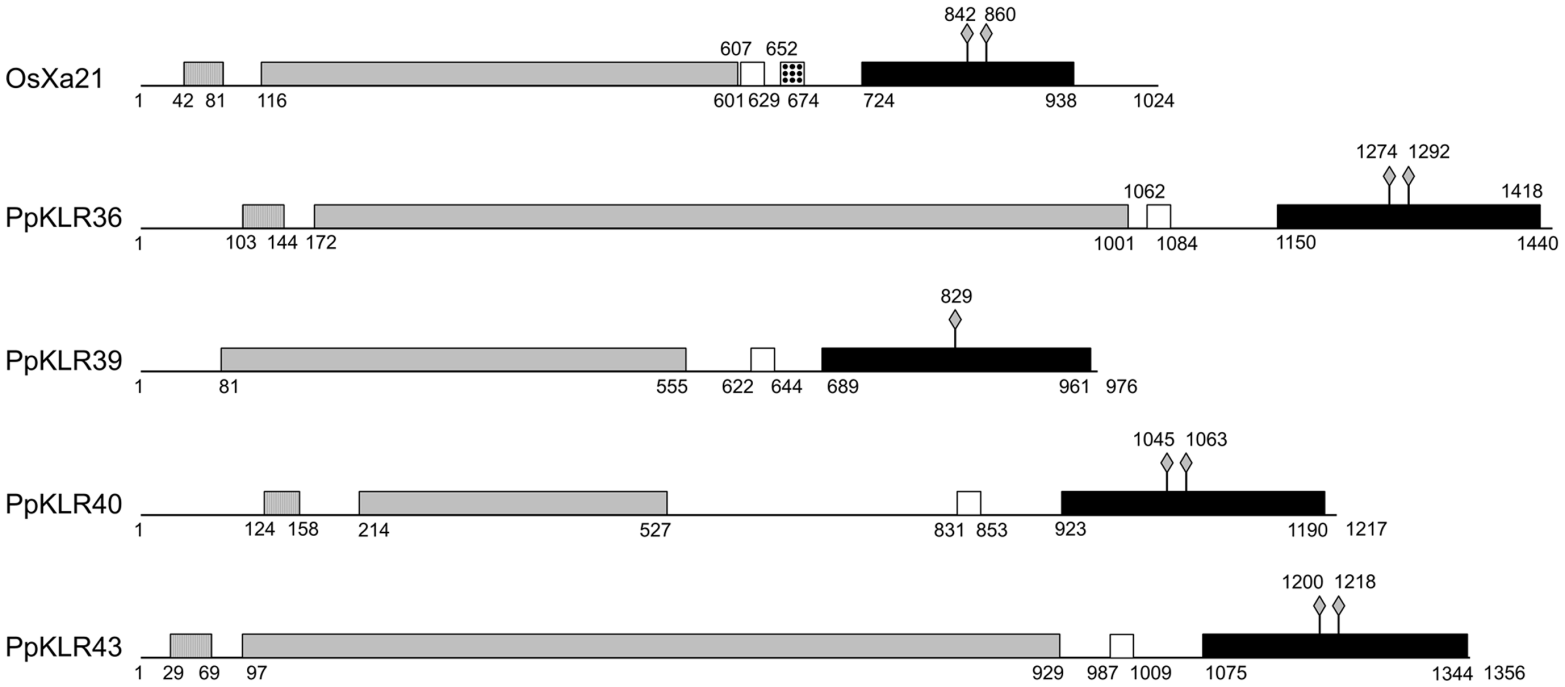
Comparison of the predicted structures of the four PpKLRs with OsXa21. Numbers indicate the putative amino acid distance from the predicted start codon. The striped, gray, white, and black boxes indicate predicted LRR N-terminal, LRR, transmembrane, and kinase domains, respectively. Diamonds show predicted Ser/Thr kinase active sites (aspartic acids). The dotted box indicates the juxtamembrane domain of OsXa21.

**Figure 5:**
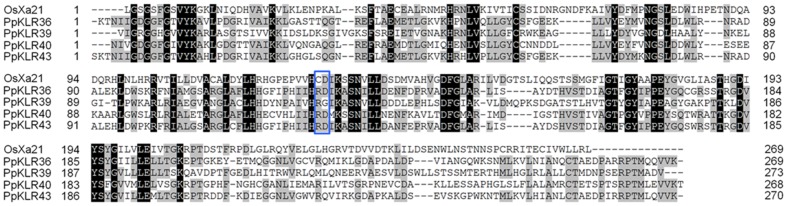
Alignment of OsXa21, PpKLR36, PpKLR39, PpKLR40, and PpKLR43 kinase domains. Amino acid sequences were obtained from the Phytozome database. The numbers indicate the distance from the start of the putative kinase domain regions. Shaded residues indicate similar amino acids: black and gray shading indicates identical and loosely identical residues, respectively. The blue box shows the predicted RD kinase characteristic region [6].

We compared the predicted structures of PpKLR36, PpKLR39, PpKLR40, and PpKLR43 with OsXa21. The conserved Ser/Thr kinase active site was observed in all four PpKLRs: D1274 and D1292 for PpKLR36, D829 for PpKLR39, D1045 and D1063 for PpKLR40, and D1200 and D1218 for PpKLR43 (Figure 4). The kinase domains of PpKLR36, PpKLR39, PpKLR40, and PpKLR43 shared 74%, 86%, 84%, and 76% similarity with the kinase domain of OsXa21 in their amino acid sequences. In addition, PpKLR39 was predicted to be a so-called non-RD kinase [10] like OsXa21 (Figure 5). The predicted maximum length of the LRR domains of the PpKLRs varied significantly. The lengths of the LRR domains of PpKLR36, PpKLR43, and PpKLR40 were ca. 400–900 aa. The full-length and LRR region of PpKLR39 were predicted to be 976 and 475 aa, respectively (Figure 4); these lengths were similar to those of OsXa21. However, PpKLR39 did not have the LRR N-terminal motif that was observed in OsXa21 and the other three PpKLRs mentioned above. All four of these PpKLRs had a predicted transmembrane domain.

### *P. patens* has PpKLR-interacting proteins

**Figure 6:**
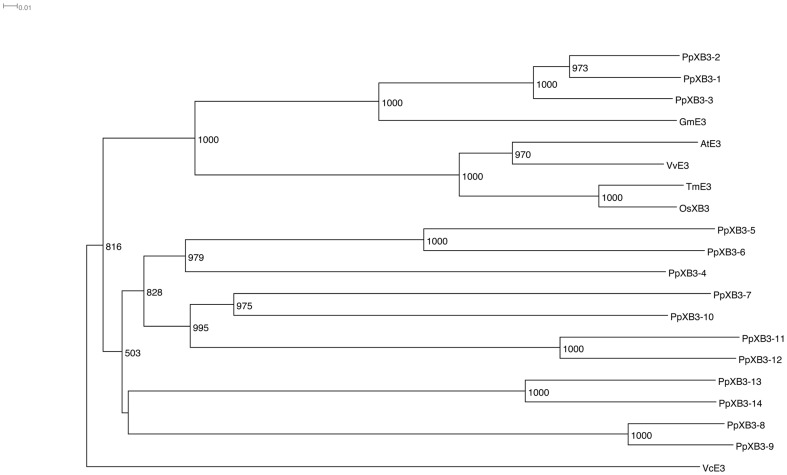
Phylogenetic analysis of PpXB3s and E3 proteins of other plants. Sequence names correspond to E3 protein sequences listed in Table S4. Phylogenetic trees were constructed by the neighbor-joining method. Numbers at the nodes indicate bootstrap values from 1,000 replicates.

**Figure 7:**
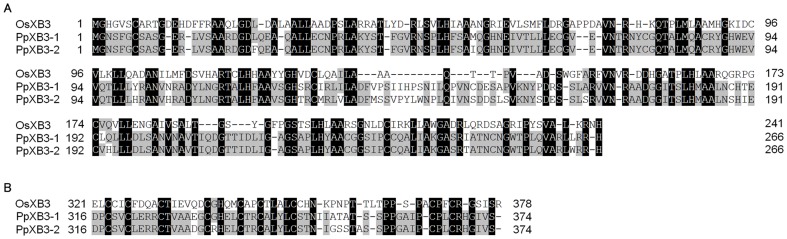
Alignment of OsXB3 and PpXB3-1 and -2. Amino acid sequences were obtained from the Phytozome database. The numbers indicate position from the putative start codon. Shaded residues indicate similar amino acids: black and gray shading indicates identical and similar residues, respectively. (A) Alignment of putative Ankyrin repeat domains. (B) Alignment of putative zinc finger domains.

Because OsXa21 was reported to interact with Xa21-binding protein 3 (XB3), which has an ankyrin repeat and a zinc finger domain, we attempted to search for XB3 homologs in the *P. patens* genome. We found that *P. patens* had at least 14 XB3 homologs (PpXB3s) (Table 4). PpXB3-1, PpXB3-2 and PpXB3-3 were predicted to have highly conserved ankyrin repeat and zinc finger domains. In phylogenetic analysis, three big clusters were suggested. One of the clusters included PpXB3-1, PpXB3-2, PpXB3-3, *Glycine max* E3 (GmE3, XP_003556093), *A. thaliana* E3 (AtE3, NP_180450), *Vitis vinifera* E3 (VvE3, XP_002283965), *Triticum monococcum* E3 (TmE3, AGH18690) and OsXB3 (Figure 6). High similarity between PpXB3-1 and PpXB3-2 was also observed in both the putative ankyrin repeat and zinc finger domains (Figure 7).

**Table 4:** Locations of PpXB3 genes in the *P. patens* genome.

	Scaffold	Location			Gene ID
*PpXB3-1*	13	146040	-	151871	Pp1s13_27V6.1
*PpXB3-2*	55	925050	-	930635	Pp1s55_144V6.1
*PpXB3-3*	256	245844	-	250491	Pp1s256_42V6.1
*PpXB3-4*	325	196917	-	200229	Pp1s325_36V6.1
*PpXB3-5*	334	405014	-	410942	Pp1s334_81V6.1
*PpXB3-6*	335	156376	-	163283	Pp1s335_34V6.1
*PpXB3-7*	32	767300	-	769123	Pp1s32_124V6.1
*PpXB3-8*	70	1144035	-	1150387	Pp1s70_186V6.2
*PpXB3-9*	143	834497	-	839838	Pp1s143_115V6.1
*PpXB3-10*	14	2788528	-	2789352	Pp1s14_440V6.1
*PpXB3-11*	10	2999427	-	3003020	Pp1s10_383V6.1
*PpXB3-12*	432	102339	-	105483	Pp1s432_19V6.1
*PpXB3-13*	6	1705592	-	1711465	Pp1s6_158V6.1
*PpXB3-14*	60	1219662	-	1225189	Pp1s60_201V6.1

Scaffold and genomic numbers for *XB3* genes in *P. patens* were obtained from the Phytozome database (Ver 9.1).

**Figure 8:**
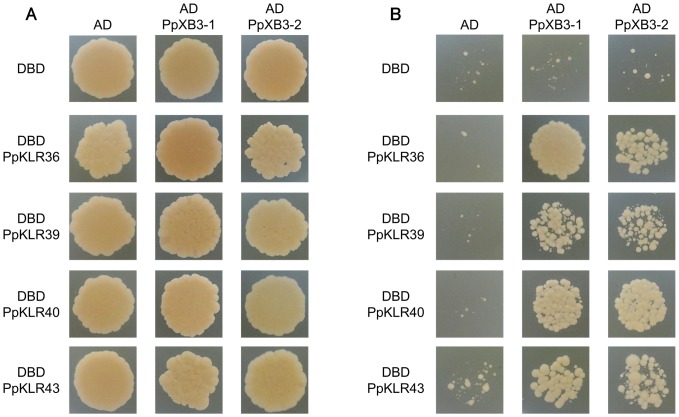
Yeast two hybrid analysis of PpKLR36, PpKLR39, PpKLR40, and PpKLR43 (AD) with PpXB3-1 and PpXB3-2 (DBD). Y2H analysis of PpXB3-1 with PpKLR36, PpKLR39, PpKLR40, and PpKLR43 was performed three times and produced the same results. This figure shows a representative example. PpKLR kinases and PpXB3s were expressed with the activation domain (AD) and DNA-binding domain (DBD) in yeast, respectively. Detection of the interactions and elimination of false positive results required that the test was made more stringent by application of 40 mM 3-AT in the culture medium. (A) Yeast cells were spotted on SD-W, L, H medium plates. (B) Yeast cells were spotted on SD-W, L, H medium with 40 mM 3-AT plates.

In rice, OsXa21 interacts with OsXB3 and transduces signals [14]. To investigate whether the PpXB3s could interact with PpKLRs, we performed yeast two hybrid analysis (Figure 8). In this experiment, the kinase domains of *PpKLR36*, *PpKLR39*, *PpKLR40*, and *PpKLR43* were inserted into the pGBT10 vector (Prey) and *PpXB3-1* and *PpXB3-2* were inserted into the pGAD9 vector (Bait). In SD-W, L, H medium without 3-AT, yeast growth was observed for all of the combinations. On 40 mM 3-AT medium, the AD (empty vector) and DBD (empty vector), AD and DBD-PpKLRs, AD-PpXBs and DBD combinations produced small colonies, whereas strong growth was observed for the DBD-PpKLR36, PpKLR39, PpKLR40, PpKLR43 and AD-PpXB3-1, PpXB3-2 combinations. These results indicate that PpKLR36, PpKLR39, PpKLR40 and PpKLR43 interact with PpXB3-1 and PpXB3-2.

## Discussion

In this study, we searched for *NL*- and *KLR*-type *R* gene homologs in the *P. patens* genome. We found that *P. patens* had 17 *kinase*-*NBS*-*LRR* and four *TIR*-*NBS*-*LRR*-type *R* gene homologs. In comparison, Xue *et al.* [23] indicated that *P. patens* has six *kinase*-*NLs*. This discrepancy could be a result of differences in prediction procedures, because we searched for individual domains using a *kinase*-*NL* sequence (*PpC24* and *LRR*) as the query and then compared distances between them. Three out of the four *TIR*-*NL*-type homologs have tetratricopeptide repeats or a zinc finger domain in the C-terminal region. Until now, *TIR-NL-*type *R* genes that have this C-terminus structure have not been reported. However, the remaining one has a premature stop codon at the C-terminus of the NBS. We also found that *P. patens* has 11 KLR-like genes in the genome. Their kinase domains all show high sequence similarity to plant Ser/Thr kinases. In addition, there is a possibility that each of the *PpTNLs*, *PpKNLs*, and *PpKLRs* has several splicing variants, especially in the LRR region. Figure S1 shows the splicing pattern that was detected in *PpKNL6* (Pp1s17_234V6.1), but it may be difficult to determine all of the potential LRR variants because of the significant repetition.

In particular, we found four putative PpKLRs (PpKLR36, PpKLR39, PpKLR40, and PpKLR43) that have high similarity to OsXa21. Because all four of these PpKLRs have TM domains, they could be membrane-anchored proteins. We also found 14 homologs of OsXB3, which has E3 ubiquitin ligase activity and interacts with Xa21 in rice. Among these, at least two (PpXB3-1 and PpXB3-2) can interact with the four PpKLRs. Our findings indicate that *P. patens* could have a rice Xa21-like KLR-type sensing system to detect specific molecules, but the target molecules are still unknown.

In the phylogenetic tree, the four PpKLRs are located near the branch for Pto [28] of *Solanum lycopersicum*. Akita and Valkonen (2002) reported that the kinase region of PpC24 was similar to that of Pto. However, the putative kinase domains of the four PpKLRs are more similar to Pto than PpC24 (74%, 76%, 72%, 75%, and 63% for PpKLR36, PpKLR39, PpKLR40, PpKLR43, and PpC24, respectively). In a BLASTP analysis of the kinase domains of the four PpKLRs, higher similarity was only observed against receptor kinases that were classified as kinase-LRR type. Although the four PpKLRs have conserved kinase motifs and predicted kinase active sites aside from the RD region, we did not detect clear kinase activity for the cloned domain (Figure S2). The activity of membrane-bound kinases has been reported to be facilitated by their juxtamembrane domains [29], [8]. OsXa21 actually has a juxtamembrane domain (22 aa in length), but none of the four PpKLRs were predicted to have such a domain. These results suggest that the four PpKLRs we have discussed above may have no kinase activity by themselves. Further research will be needed to determine whether the PpKLRs have kinase activity with other proteins such as PpXB3, since some receptor kinases, e.g. BRI1 [30], are reported to require specific proteins for catalysis.

We searched the whole genome sequence of *P. patens* and found *NL-* and *KLR-*type *R* gene candidates. In particular, we identified the possible existence of KLR-type receptor-like kinases (PpKLRs) and their interacting proteins (PpXB3s). It has been demonstrated that KLR proteins are involved in a broad range of biological processes such as responses to environmental stress. However, information on KLRs in *P. patens* is currently quite limited (e.g., [31]). Our findings will contribute to analyzing the functions of receptor-like kinases in plants because of the valuable properties of *P. patens* for molecular biology. Further research will be required to identify what molecules are specific ligands for the PpKLRs and also to determine the biological functions of the PpKLRs and PpXB3s.

## Supporting Information

Figure S1 **Primary structure of KNL6.**
(DOC)

Figure S2 **Expression of kinase and kinase activity of PpKLR39 and PpKLR40.**
(DOC)

Table S1 **Primers for analysis and cloning of PpKLRs.**
(DOC)

Table S2 **Genomic locations of 29 predicted kinases.**
(DOC)

Table S3 **Genes uesd for phylogenetic analysis of the** ***P. patens*** **kinases.**
(DOC)

Table S4 **E3 proteins of other plants used for phylogenetic analysis.**
(DOC)
