# Supplementary material for: Physcomitrella patens Has Kinase-LRR R Gene Homologs and Interacting Proteins
Source: PLoS One. 2014 Apr 18;9(4):e95118. doi: 10.1371/journal.pone.0095118 (PMC3991678; doi:10.1371/journal.pone.0095118)
Supplement: Figure S1 — Primary structure of KNL6. (DOC) [file pone.0095118.s001.doc]

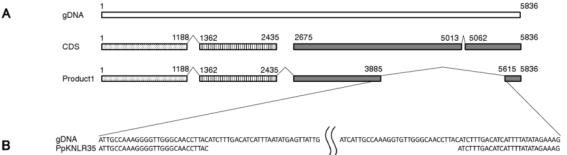


Figure S1. Primary structure of KNL6

The CDS predicted in Phytozome and our cloned KNL6 (Product 1, 3693 bp) are compared. The full length of KNL6 was cloned using RT-PCR products. Primers were designed from the sequence in the Phytozome database. Note that the CDS corresponding to KNL6 in Phytozome is registered into two genes. We also detected another length of cDNA (3481 bp); the splicing position is difficult to determine because of the significant repetition. Dotted, striped, and gray boxes indicate kinase, NBS, and LRR regions.
