# Supplementary material for: Physcomitrella patens Has Kinase-LRR R Gene Homologs and Interacting Proteins
Source: PLoS One. 2014 Apr 18;9(4):e95118. doi: 10.1371/journal.pone.0095118 (PMC3991678; doi:10.1371/journal.pone.0095118)
Supplement: Figure S2 — Expression of kinase and kinase activity of PpKLR39 and PpKLR40. (DOC) [file pone.0095118.s002.doc]

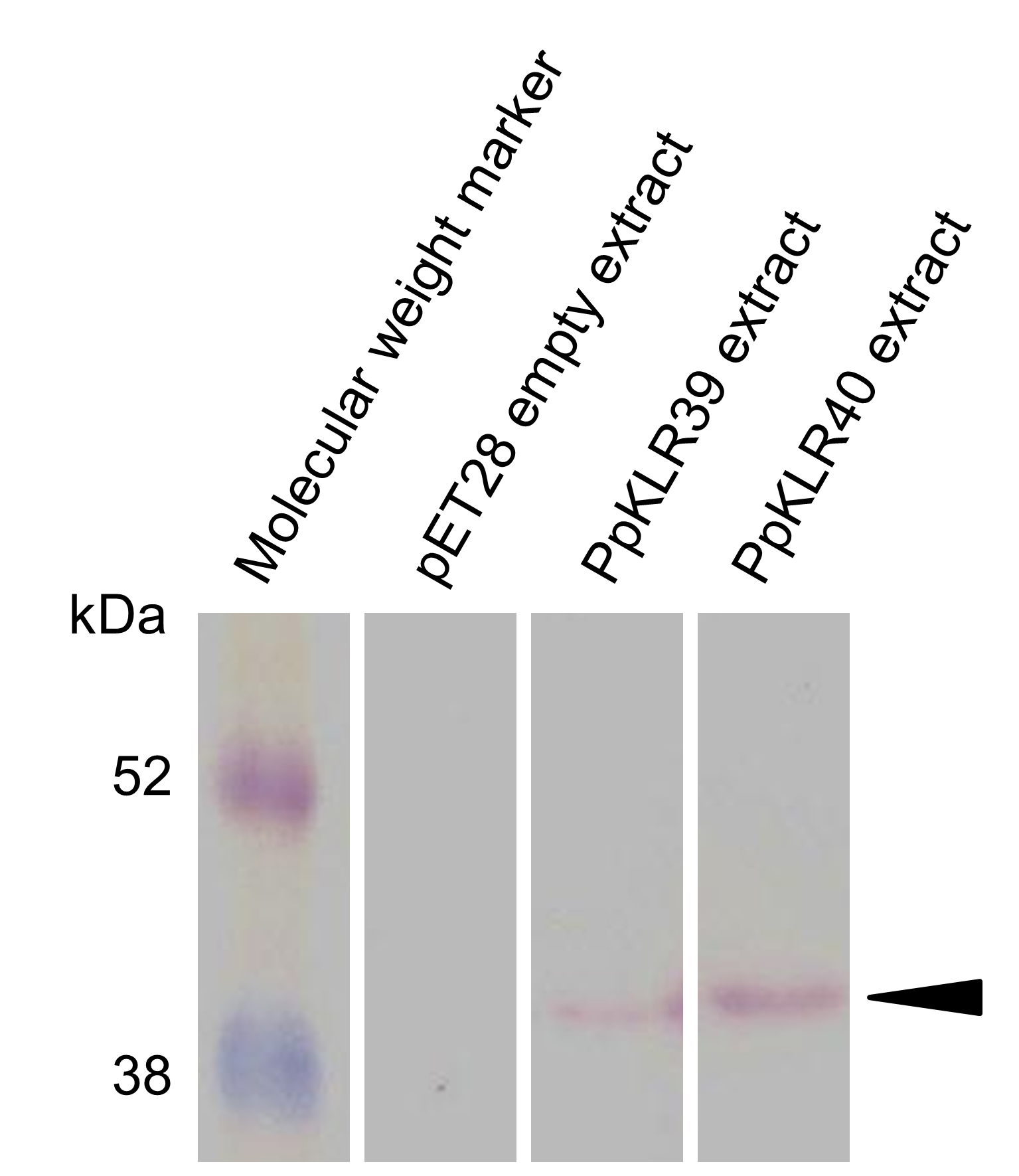
A

B


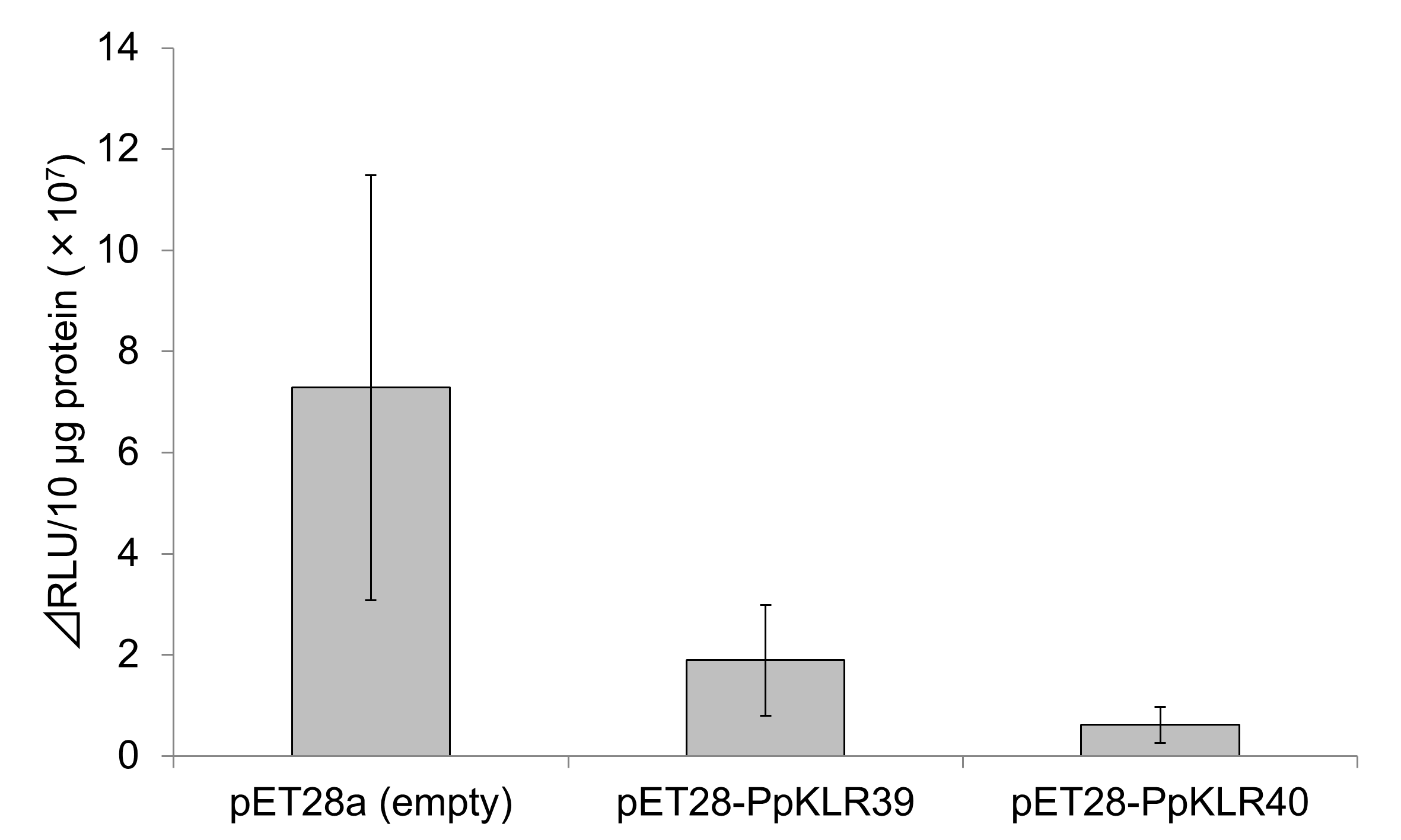


Figure S2. Expression of kinase and kinase activity of PpKLR39 and PpKLR40

cDNAs of the predicted kinase domains of the PpKLR39 (1375684-965 aa) and PpKLR40 (920-1190aa) genes were cloned into the HincII site of the pBlueScript SK plasmid using Mighty Mix (Takara). Primers were designed according to sequence data from the Phytozome database (see Table S1). The plasmids were digested with KpnI/EcoRV and the fragments were cloned into the pET28a (+) vector at the SacI site. pET28-PpKLR39 and 40 were transferred into the *E. coli* *Rosetta de3* strain. The *Rosetta* pET28-PpKLR39 and pET28-PpKLR40 were grown in 50 or 250 mL of Terrific medium supplemented with 50 μg/mL ampicillin to A_600_ = 0.6–1.0. For expression of the fusion proteins, 0.25 mM isopropyl-1-thio-β-d-galactopyranoside was added and cultured for 12 h at 18°C. The cells were collected by centrifugation (20,000 × g) for 10 min at 4°C. The pellets were washed once with 0.9% NaCl and collected by centrifugation (20,000 × g) for 10 min at 4°C. The pellets were resuspended in 5 mL buffer (10 mM KH_2_PO_4_ and 5 mM EDTA, pH 7.0) and lysed by sonication on ice. The supernatant and pellet were collected by centrifugation (20,000 × g) for 10 min at 4°C. Because the recombinant protein was insoluble in the pET28-PpKLR40 transformant, the pellet was dissolved in 6 M guanidine hydrochloride for 1 h at 25°C and then the lysate was dialyzed using a membrane (12000-14000 MWCO; Fisher Scientific, Waltham, MA, USA) and 50 mM HEPES (pH 7.0) as a buffer. The dialysis buffer was exchanged once at 9 h, and again at 15 h of dialysis. The dialysate was enriched by ultrafiltration (10,000 NMWL; Billerica, MA, Millipore). The concentration of proteins was measured by the Bradford protein assay. The proteins and Molecular weight marker (General Electric, Fairfield, CT, USA) were separated by 10% SDS-PAGE gels and transferred to PVDF membrane (pore size 0.2 μm, Port Washington, NY, USA, Pall) by electro-blotting. First antibodies were targeted to His tag and second antibodies were labeled with alkaline phosphatase. Immunoblot detection was performed using Western Blue Stabilized Substrate for alkaline phosphatase (Promega). Kinase activity was measured using Kinase-Gol Luminescent Kinase Assays (Promega). The kit are homogeneous non-radioactive methods for determining the activity of kinases by quantifying the amount of ATP remaining in solution following a kinase reaction. The signal was detected for 5 s with a Gene Light GL-200 (MICROTEC, Urayasu, Japan). The reaction solution contained 100 mM HEPES (pH 7.0), 2 mM MgCl_2_, 1 mM dithiothreitol, 20 μM ATP, 10 μM AMP, 10 μg casein (substrate) and 10 μg sample. This sample was not purified. ΔRelative Light Unit (ΔRLU) indicate the difference of chemiluminescence from reaction solution without protein sample. (A) Expression of PpKLR39 and PpKLR40 kinase domain detected by western blotting. Arrow indicates the corresponding size. (B) Kinase activity of cloned PpKLR39 and PpKLR40 kinase. Activity of empty vector may indicate the endogenous activity of *E. col*. Since expressed protein was added to the background, relative activity per total protein could be decreased if the expressed protein has no kinase activity. ΔRelative Light Unit (ΔRLU)
