# Supplementary material for: Physcomitrella patens Has Kinase-LRR R Gene Homologs and Interacting Proteins
Source: PLoS One. 2014 Apr 18;9(4):e95118. doi: 10.1371/journal.pone.0095118 (PMC3991678; doi:10.1371/journal.pone.0095118)
Supplement: Table S1 — Primers for analysis and cloning of PpKLRs. (DOC) [file pone.0095118.s003.doc]

Table S1. Primers for analysis and cloning of PpKLRs

| Gene | Target domain | F/R^1^ | Sequence |
| --- | --- | --- | --- |
| PpKLR36 | kinase-LRR^2^ | F | catgggagacttccgctcag |
|  |  | R | cccgacggtaggattgtagc |
|  | Kinase^3^ | F | cgacggtaggattgtagccatcaag |
|  |  | R | cagcatcttcaccacttgttgcatg |
|  |  |  |  |
| PpKLR39 | kinase-LRR^2^ | F | cactcatggcactcaagcca |
|  |  | R | agggggtagcaaacggattg |
|  | kinase ^3^ | F | cctaagtgatagttgcgtaattggaag |
|  |  | R | taaaattccgaccacatctgccattg |
|  |  |  |  |
| PpKLR40 | kinase-LRR^2^ | F | gcgtttgcttccgtccattt |
|  |  | R | cgaaatggaggaagcgcatg |
|  | kinase^3^ | F | tagtccggaaaacatagttggagatg |
|  |  | R | tagcgtttttacaacctcaagcatcgtg |
|  |  |  |  |
| PpKLR43 | kinase-LRR^2^ | F | atttctgggacggtctcgtg |
|  |  | R | gacaaacatcatcggcgacg |
|  | kinase^3^ | F | cttcagcaagacaaacatcatcggcga |
|  |  | R | ctatgcttcaagtggtgaagtttctca |

^1^ F: Forward, R: Reverse.

^2^ Primers for gene expression analysis.

^3^ Primers for yeast two hybrid and kinase activity analysis.
