# Supplementary material for: Physcomitrella patens Has Kinase-LRR R Gene Homologs and Interacting Proteins
Source: PLoS One. 2014 Apr 18;9(4):e95118. doi: 10.1371/journal.pone.0095118 (PMC3991678; doi:10.1371/journal.pone.0095118)
Supplement: Table S2 — Genomic locations of 29 predicted kinases. (DOC) [file pone.0095118.s004.doc]

Table S2. Genomic locations of 29 predicted kinases

|  | Scaffold | Location | | | Locus name |
| --- | --- | --- | --- | --- | --- |
| 1 | 1 | 2232190 | - | 2232873 | BAD38895 |
| 2 | 107 | 730620 | - | 731783 | Pp1s107_101V6 |
| 3 | 113 | 997,786 | - | 996,970 | Pp1s113_185V6 |
| 4 | 124 | 497,398 | - | 496,631 | Pp1s124_58V6 |
| 5 | 131 | 362,408 | - | 363,245 | XP_001773049 |
| 6 | 158 | 673,539 | - | 674,247 | Pp1s158_132V6 |
| 7 | 158 | 701,914 | - | 702,130 | Pp1s158_140V6 |
| 8 | 17 | 342,232 | - | 341,440 | Pp1s17_81V6 |
| 9 | 17 | 1,531,806 | - | 1,530,990 | Pp1s17_234V6 |
| 10 | 17 | 1840202 | - | 1841041 | Pp1s17_276V6 |
| 11 | 180 | 434,625 | - | 435,333 | Pp1s180_59V6 |
| 12 | 180 | 593,825 | - | 593,270 | Pp1s180_84V6 |
| 13 | 180 | 691,191 | - | 690,591 | Pp1s180_101V6 |
| 14 | 2 | 1242958 | - | 1243644 | Pp1s2_244V6 |
| 15 | 2 | 1,442,620 | - | 1,441,788 | Pp1s2_293V6 |
| 16 | 223 | 481380 | - | 482156 | Pp1s223_84V6 |
| 17 | 241 | 111,923 | - | 112,454 | XP_001755370 |
| 18 | 278 | 161,414 | - | 162,167 | XP_001785719 |
| 19 | 345 | 337089 | - | 337559 | Pp1s345_31V6 |
| 20 | 35 | 627333 | - | 628133 | Pp1s35_121V6 |
| 21 | 358 | 292,800 | - | 292,210 | Phpat.002G003500 |
| 22 | 37 | 146,930 | - | 147,146 | Pp1s37_18V6 |
| 23 | 37 | 328,596 | - | 328,041 | Pp1s37_60V6 |
| 24 | 396 | 166,075 | - | 166,645 | Pp1s396_25V6 |
| 25 | 40 | 1047528 | - | 1,047,999 | Pp1s40_165V6 |
| 26 | 44 | 590,532 | - | 589,682 | Phpat.021G055200 |
| 27 | 508 | 6,489 | - | 5,694 | Pp1s508_5V6 |
| 28 | 68 | 289,607 | - | 288,798 | Pp1s68_70V6 |
| 29 | 7 | 124197 | - | 125120 | Pp1s7_28V6 |

Scaffold and location numbers were obtained from the Phytozome database (v 9.1).
