# Supplementary material for: Physcomitrella patens Has Kinase-LRR R Gene Homologs and Interacting Proteins
Source: PLoS One. 2014 Apr 18;9(4):e95118. doi: 10.1371/journal.pone.0095118 (PMC3991678; doi:10.1371/journal.pone.0095118)
Supplement: Table S3 — Genes uesd for phylogenetic analysis of the P. patens kinases. (DOC) [file pone.0095118.s005.doc]

Table S3. Genes uesd for phylogenetic analysis of the P. patens kinases

| Name | Origin | Accession Number |
| --- | --- | --- |
| AtS6K | *Arabidopsis thaliana* | NP_187485 |
| PpS6K | *P. patens* | AEW26787 |
| PpNPL1 | *P. patens* | NP_851210 |
| PpPDK | *P. patens* | NP_568138 |
| Pto | *Solanum lycopersicum* | AAZ15370 |
| PpRAPTOR1 | *P. patens* | NP_566335 |
| At Ser/Thr kinase | *Arabidopsis thaliana* | NP_568893 |
| Cr Tyr kinase | *Chlamydomonas reinhardtii* | XP_001691230 |
| At His kinase | *Arabidopsis thaliana* | XP_002983016 |
| Zm His kinase | *Zea mays* | NP_56527 |
| At Tyr kinase | *Arabidopsis thaliana* | AAL58946 |

Sequences were obtained from the NCBI database.
