# Supplementary material for: Physcomitrella patens Has Kinase-LRR R Gene Homologs and Interacting Proteins
Source: PLoS One. 2014 Apr 18;9(4):e95118. doi: 10.1371/journal.pone.0095118 (PMC3991678; doi:10.1371/journal.pone.0095118)
Supplement: Table S4 — E3 proteins of other plants used for phylogenetic analysis. (DOC) [file pone.0095118.s006.doc]

Table S4. E3 proteins of other plants used for phylogenetic analysis

| Name | Origin | Accession Number |
| --- | --- | --- |
| OsXB3 | *Oryza sativa* | NP_001054449 |
| AtE3 | *Arabidopsis thaliana* | NP_180450 |
| GmE3 | *Glycine max* | XP_003556093 |
| VvE3 | *Vitis vinifera* | XP_002283965 |
| TmV3 | *Triticum monococcum* | AGH18690 |
| VcE3 | *Volvox carteri* | XP_002955294 |

The E3 amino acid sequences of these plants were obtained from the NCBI database.
